# Traitement chirurgical des kystes de Tarlov sacrés: à propos de 20 cas

**DOI:** 10.11604/pamj.2019.33.98.10760

**Published:** 2019-06-10

**Authors:** Mohammed Yassine Haouas, Mohamed Khoulali, Zinelabidine En-Nhaili, Hani El-Johani, Mounir Rghioui, Robin Srour

**Affiliations:** 1 Université Hassan II, Faculté de Médecine et de Pharmacie, CHU Ibn Rochd, Service de Neurochirurgie, Casablanca, Maroc; 2 Université Mohamed I, Faculté de Médecine,CHU Mohamed VI, Service de Neurochirurgie, Oujda, Maroc; 3 Université de Strasbourg, Faculté de Médecine, CHU Haute-pierre, Neurochirurgie, Strasbourg, France; 4 Hôpitaux Civils de Colmar, Hôpital Pasteur, Service de Neurochirurgie, Colmar, France

**Keywords:** Kyste de Tarlov, kyste périneural sacré, diverticule de la racine nerveuse, ponction du kyste, traitement chirurgical, manchon de la dure mère, Tarlov cyst, perineural sacral cyst, nerve root diverticulum, cyst aspiration, surgical treatment, dural sheat

## Abstract

Le kyste de Tarlov ou kyste périneural est une dilatation locale de l'espace sous-arachnoïdien se formant au contact d'une racine nerveuse et est rempli de liquide cérébro-spinal. Il n'y a pas de consensus sur le traitement optimal des kystes périnerveux sacrés symptomatiques. De nombreuses méthodes ont été appliquées pour traiter ces lésions symptomatiques, avec des résultats variables. Nous rapportons une série de 20 patients opérés pour un kyste de tarlov sacré. Nos résultats étaient satisfaisants avec 80% d'amélioration et sans aggravation neurologique en post-opératoire. Notre technique chirurgicale (laminectomie sacrée + ponction du kyste + mise en place d'un manchon dure-mèrien), décrite pour la première fois dans ce travail, semble efficace dans les 20 cas rapportés dans notre série.

## Abstract (en)

Tarlov cyst or perineural cyst is a local dilation of the subarachnoid space formed within the nerve root and filled with cerebrospinal fluid. There is no consensus on the best treatment of syntomatic sacral perinervous cysts. Many methods have been used to treat these symptomatic lesions, with variable results. We report a case series including 20 patients undergoing surgery for sacral Tarlov cyst. Our results were satisfactory; 80% of patients improved without neurological worsening in the post-operative period. Our surgical technique (sacral laminectomy+cyst puncture+establishment of dural sheat) described for the first time in this study seems to have been effective in the 20 cases reported in our study.

## Introduction

Un kyste de Tarlov ou kyste périneural est une dilatation locale de l'espace sous-arachnoïdien se formant au contact d'une racine nerveuse et est rempli de liquide cérébro-spinal. Les kystes de Tarlov concernent le plus souvent les racines sacrées, on doit leur première description en 1938, au neurochirurgien américain Isadore Tarlov. Ces lésions sont souvent de découverte fortuite sur l'imagerie par résonance magnétique (IRM), et la plupart d'entre eux sont asymptomatiques [1]. Dans une série de 500 IRM consécutives de la colonne lombo-sacrée, Paulsen *et al.* [2] a enregistré une incidence de 4,6%, dont 20% des kystes qui étaient symptomatiques. Environ 1% des kystes périnerveux sacrés devienent symptômatiques et la cause est liée à la compression locale de la racine nerveuse [2]. L'origine de ces lésions est controversé et peu claire, avec des preuves de causalité de l'inflammation dans l'espace sous-arachnoïdien, hémorragie traumatique ou pseudomeningocèles, les diverticules congénitales, des fissures embryonnaires persistants, ou les anomalies des pressions hydrostatiques du liquide céphalorachidien [3-8]. Les douleurs périnéales ou pelviennes, les troubles urinaires sont l'apanage des femmes. Les hommes présentent essentiellement des lombosciatalgies. Il n'y a pas de consensus sur le traitement optimal des kystes périnerveux sacrés symptomatiques [9]. De nombreuses méthodes ont été appliquées pour traiter ces lésions symptomatiques, avec des résultats variables. Le drainage lombaire du LCS, le shunt lombopéritonéal et le shunt du kyste méningé ne sont pas efficaces comme traitement pour les kystes de Tarlov symptomatiques [1, 10]. La ponction percutanée scano-guidée du kyste avec une perfusion de la colle de fibrine a abouti à des résultats mitigés, et cette méthode a été associée à un taux élevé de méningite aseptique [2]. De nombreuses techniques chirurgicales ont été utilisées pour traiter ces lésions symptomatiques, avec des résultats variables. Dans ce travail nous présentons vingt cas de kystes de Tarlov traités par chirurgie (laminéctomie,ponction du kyste avec une aiguille fine et un cerclage avec un manchon dure-merien).

## Méthodes

**Figure 1:**
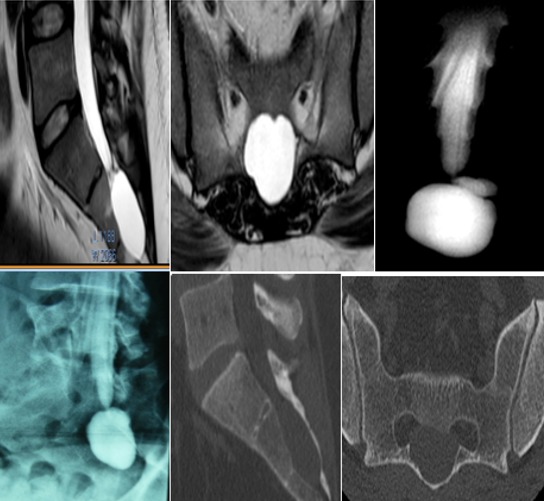
patient 35 ans, clinique: lombo-sciatalgies bilaterales avec irradiations inguino-scrotale évoluant depuis six mois, pas de troubles sphinctériens, examen normal des membres inférieurs et de la région périnéale; IRM T2 coupe sagittale et axiale montrant une masse hyperintense comme le LCS, la radiculosagraphie montre un remplissage rapide du kyste; le scanner ne montre pas une effraction osseuse

Entre janvier 2013 et décembre 2015, 20 patients (2 hommes, 18 femmes) allant de 35 à 81 ans (moyenne 61,85 ans) ont été opérés au service de neurochirurgie à l'hôpital Pasteur, Colmar, France. Le suivi obtenu à partir des visites de retour à l'hôpital ou par des entrevues téléphoniques. Tous les patients avaient un examen neurologique complet, une IRM préopératoire pour définir la localisation, la taille et le nombre des kystes, un scanner avec des fenêtres osseuses pour chercher une érosion ou une hypoplasie osseuse au voisinage du kyste et une radiculosaccographie préopératoire pour mettre en évidence le caractère communicant ou non du kyste (Figure 1). Ces éléments radiologiques nous paraissent fondamentaux dans la conduite thérapeutique des kystes péri-neuraux (Tableau 1).

**Tableau 1:** les caractéristiques et présentation clinique des patients

Nombre des patients	Age	sexe	Début des symptômes	localisation	sciatalgies	Troubles urinaires	Douleurs périnéales	Déficit moteur	Hypoesthésie vaginale
1	68	F	7 mois	S1S2	oui	Oui	Oui	Oui	non
2	43	F	5 mois	S3	non	Non	Oui	Non	non
3	57	F	11 mois	S3	non	Oui	Oui	Non	non
4	51	F	8 mois	S2	Non	Oui	oui	Non	non
5	56	F	1 an	S1	Oui	Non	Non	Non	non
6	76	F	1 an et demi	S2S3	Oui	non	non	Non	non
7	62	F	7 mois	S2	oui	oui	oui	Non	oui
8	67	F	1 an et 8 mois	S2S3	Non	oui	Oui	Non	non
9	55	F	6 mois	S2	Non	Oui	Oui	Non	non
10	61	F	9 mois	S2	Oui	Non	Non	Non	non
11	75	F	1 an et 2 mois	S1S2	Non	Oui	Oui	Non	non
12	73	F	3 ans et demi	S2S3	Non	non	Oui	Non	non
13	35	H	6 mois	S2	Oui	Non	Oui	Non	non
14	58	F	8 mois	S3	Non	Oui	Oui	Non	non
15	70	F	1 an et 4 mois	S2	Non	Non	Oui	Non	non
16	67	M	7 mois	S2	Oui	Non	Oui	Non	non
17	45	F	14 mois	S3	Non	Oui	Oui	Non	non
18	81	F	2 ans et demi	S2	Oui	Oui	Oui	Non	non
19	70	F	18 mois	S2	Non	Non	Oui	Non	non
20	67	F	10 mois	S1S2	Oui	Non	Non	Non	non

## Résultats

**Figure 2:**
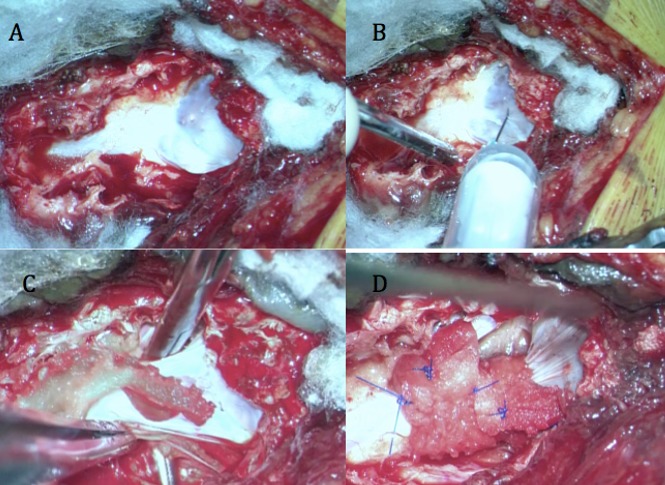
montre notre technique chirurgicale chez le même patient: A) laminectomie et mise en evidence du kyste; B) ponction du kyste à l´aide d´une aiguille fine; C) dissection du kyste; D) mise en place d´un dure-merien

**Figure 3:**
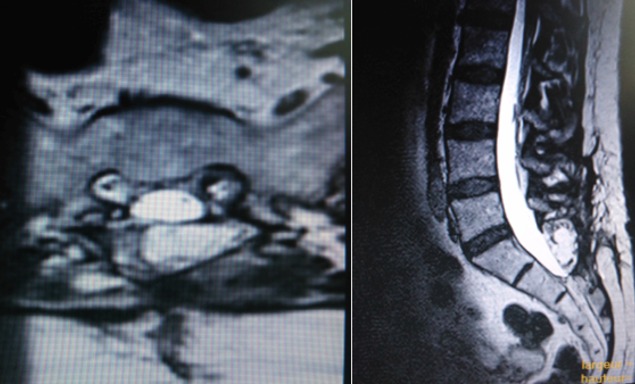
IRM de contrôle en coupes T2 axiale et sagittale qui montre l´affaissement du kyste

Dans notre série, une nette dominance de patients de sexe féminin a été observée, comme dans la plupart des études publiées dans la littérature [11]. La durée moyenne de l'histoire avant le diagnostic était de 8,4 mois (intervalle de 5 mois à 3 ans et demi). La plupart des patients avaient subi une thérapie physique et un traitement médical (médicamenteux anti-inflammatoire stéroïdien stéroïdiens et non) sans amélioration clinique. Un seul cas avait subi une ponction scanoguidée de la colle de fibrine. Les symptômes préopératoires étaient la douleur péri-anale et périnéale dans 16 cas, les sciatalgies dans 9 cas, les troubles urinaires dans 10 cas, un déficit moteur dans un cas, et une patiente qui a rapporté une hypoesthésie vaginale. 12 patients ont rapporté des douleurs croissantes quand ils ont exécuté des manoeuvres de Valsalva. Le niveau vertébral des kystes était S1 dans un cas, S1-S2 dans trois cas, S2-S3 dans trois cas, S2 dans neuf cas, S3 dans quatre cas. Chez 12 patients il y avait des kystes multiples: traitement a été choisi en fonction des kyste symptomatiques (Tableau 1). Le traitement chirurgical a été indiqué dans le cas des kystes communicants, et aussi pour les kystes non communicants sans érosion ou une hypoplasie osseuse en plus des kystes même avec une érosion osseuse mais après échec de la ponction injection de la colle, avec une étude électrophysiologique concordante dans tous les cas (Figure 1). Concernant notre technique chirurgicale (Figure 2): tous nos patients ont été opérés par une laminectomie avec une ponction du kyste à l'aide d'une aiguille fine et la mise en place d'un manchon dure-mérien. Après la chirurgie on note une amélioration significative chez seize patients (80%) avec EVA entre 0 et 4, et avec une IRM post-opératoire satisfaisante (Figure 3). Quatre patients (20%) sont restés stationnaires sans aucune amélioration, et on n'a pas eu d'aggravation neurologique dans les suites post opératoires. Ce qui concerne les troubles urinaires, on note une amélioration chez quatre patientes sur neuf.

## Discussion

Les kystes de Tarlov périnerveux ou les diverticules de la racine nerveuse sont le type II dans la classification des kystes méningés: leurs murs peuvent contenir des fibres nerveuses [12]. Les kystes méningés sont classés à la fois en fonction de leur situation par rapport à la dure-mère et l'espace sous arachnoïdien et selon la pathologie de la paroi du kyste [13]. L'incidence de kystes périnerveux sacrés a été estimée à 1,5% à 4,6%, mais les cas symptomatiques sont rares, représentant 1% du total [2, 14]. Acosta *et al.* [9] suggère que la plupart des kystes restent asymptomatiques pendant toute la vie du patient, l'histoire naturelle des kystes de Tarlov symptomatiques [< 1% [2, 14]] est l'élargissement progressif conduisant à l'érosion osseuse et l'apparition des symptomes. Ces kystes, quand ils sont symptomatiques, peuvent causer une variété de symptômes, un dysfonctionnement urinaire, la douleur radiculaire, et des paresthésies [9]. L'origine de ces lésions est controversée et incertaine. Tarlov [8] a découvert les cellules inflammatoires dans certaines parois des kystes et des tissus adjacents, le conduisant à postuler que ces kystes ont été formés par un processus inflammatoire dans la gaine de la racine nerveuse, suivie par l'inoculation du LCS. Une origine hémorragique a été décrit plus tard par Tarlov [6-8] et les autres [4], dans lequel la formation des kystes se produit en raison de l'infiltration du sang et c'est le cas dans l'hémorragie sous-arachnoïdienne ou une hémorragie post-traumatique, suivie par la dégénérescence de globules rouges qui détruit le tissu neural. Nishiura *et al*. [3] ont décrit une histoire de traumatisme antérieur dans 40% de leurs patients avec des kystes de Tarlov. Schreiber *et al.* [4] et Strully *et al.* [5] ont noté que les kystes peuvent se former à la suite de lacérations durales au cours de la chirurgie de la colonne vertébrale, conduisant à la formation pseudoméningocèle. Certains chercheurs préfèrent une origine congénitale et émettent l'hypothèse que ces kystes peuvent résulter d'un diverticule ou une faiblesse congénitale de la dure-mère, ou de fissures embryonnaires persistantes [15]. Les kystes de Tarlov ont été associées à d'autres anomalies congénitales comme les maladies du tissu conjonctif et les duplications de la gaine de la racine nerveuse [16, 17]. Enfin, certains chercheurs ont attribué la pression hydrostatique du CSF en tant que facteur causal dans la formation, la croissance et la symptomatologie des kystes [2, 10]. Ils ont fait valoir que la pression hydrostatique du LCS est exercée le long de l'espace sous-arachnoïdien et augmente avec des pulsations systolique ou des manœuvres de Valsalva. L'élargissement des kystes se produit par l'intermédiaire de cette action mécanique pulsatile de LCS, donc les filaments des racines sensorielles sont étirées en raison de la proximité des kystes, et sont comprimées contre l'os adjacent ou contre d'autres racines nerveuses: cela provoque des troubles sphinctériens, des douleurs, ou d'autres perturbations [2, 8, 10, 18]. Les descriptions de l'imagerie dans les publications originales ont mis en évidence que l'absence de la prise de contraste précoce sur la myélographie est une caractéristique pathognomonique des kystes de Tarlov, et permet de les distinguer des kystes extraduraux (type I), et les kystes méningés intra duraux ou les diverticules méningées (Type III) [4, 8]. Parce que dans les kystes de Tarlov, il y a une prise de contraste tardive sur la myélographie [8]. Les kystes de Tarlov ont la même densité que le LCS sur la tomodensitométrie sans injection de produit de contraste et peuvent provoquer de diverses anomalies et érosions osseuses [19]. Le myéloscanner démontre la communication entre le kyste et l'espace sous-arachnoïdien [20]. L'IRM est considéré comme l'examen radiologique de choix quand un kyste périneurale sacrée est suspectée. Ses avantages comprennent la haute résolution sur les parties molles [21, 22]. L'IRM montre généralement les mêmes caractéristiques d'intensité de signal compatibles avec les kystes contenant le LCS [23].

**Traitement**: il y a beaucoup de controverse concernant le traitement optimal des kystes de Tarlov symptomatiques. Mitra *et al.* [12] suggèrent que les stéroïdes oraux et injectés en épidural peuvent être un complément utile dans le traitement conservateur des kystes périneurales. L'efficacité de stéroïdes dans la gestion du kyste périneurale symptomatique nécessite une enquête plus approfondie car les conclusions de leurs constatations sont limitées en raison de la petite taille de l'échantillon (un kyste lombaire, un kyste cervical). Le développement et la disponibilité des scanners et des IRM modernes ont renforcé la reconnaissance de ces lésions, et, en outre, ont permis de réaliser des techniques mini-invasives tels que l'aspiration percutanée et le drainage scanno-guidé. Cependant, Paulsen *et al.* [2] ont signalé une réapparition des symptômes 3 semaines à 6 mois après drainage percutané CT-guidé dans tous les cas (cinq patients). D'autre part, Voyadzis *et al.* [17] n'a observé aucun soulagement de la douleur dans leur série (trois patients). Lee *et al.* [1] ont signalé une récurrence des symptômes après quelques jours à plusieurs semaines dans tous les cas (trois patients), mais ça reste des petites séries ou les résultats restent limités, car dans notre service de neurochirurgie le professeur Maitrot D a rapporté une amélioration chez 65 patients dans une série de 95 patients traités par ponction aspiration scanno-guidée et injection de colle à l'aide de deux aiguilles et pour le reste des patients on note qu'il y avait pas d'amélioration dans 17 cas, une aggravation transitoire dans 3 cas, et un échec de la technique chez 10 patients. Zhang *et al.* [24] ont traité 31 patients avec une ponction injection percutanée CT-guidée de colle de fibrine avec et sans aspiration du LCS, ils ont décrit une amélioration des symptômes de 80% avec aspiration et une amélioration des symptômes de 75%, sans aspiration, il n'y avait aucune récidive des kystes traités au cours d'un suivi moyen de 23 mois et ils ont rapporté deux cas de méningite aseptique transitoires. Murphy *et al.* [15] décrit rétrospectivement une plus grande série de 122 patients traités par CT guidées aspirations d'aiguilles à injection de colle de fibrine de kyste. En utilisant la technique à aiguille unique et les deux-aiguille [25] ou la technique de l'aiguille canule, les enquêteurs ont obtenu une amélioration symptomatique chez 65% des patients, bien que 23% ont connu une récidive des symptômes après 7,3 mois. Le traitement chirurgical des kystes périneurales symptomatiques qui a été préconisé par Tarlov impliquant l'ablation du kyste et l'excision complète de la racine postérieure affectée et le ganglion, et depuis, cette technique a été utilisée par d'autres chirurgiens. Cependant, certains chercheurs affirment que la morbidité résultant des complications neurologiques attribuables à cette procédure est trop grande pour justifier son utilisation, en particulier lorsqu'ils traitent les kystes sacrés multiples. Dans le tableau 2, nous avons comparé les résultats des études de la littérature des différentes techniques de microchirurgie dédiées au traitement des kystes de Tarlov [14, 16,17, 20, 21, 23, 26-30]. La laminectomie de décompression simple a été tentée, mais son taux de réussite était faible; d'autres thérapies chirurgicales comprennent la résection du kyste au col, la résection de la paroi du kyste, la fenestration et l'imbrication du kyste, et la rétraction du kyste aide d'une coagulation bipolaire (Tableau 2). Les complications rapportées dans la littérature comprennent la prostatite, le saignement au niveau de la fosse cérébrale postérieure, fuite du LCR, délogement du patch musculaire, l'incontinence urinaire, la dislocation du cathéter, douleurs persistantes, et la thrombose veineuse profonde [10, 14, 17, 23, 26, 29, 30]. Voyadzis *et al.* [17], Guo *et al.* [26], Tanaka *et al.* [30], et Neulen *et al.* [23] ont suggéré que le traitement chirurgical est indiqué pour les kystes de plus de 1 à 1,5 cm avec des symptômes radiculaires (fortement corrélés avec un excellent résultat clinique). Davis *et al.* [11] n'a trouvé aucune différence significative dans la taille entre les kystes symptomatiques et asymptomatiques (P > 0,05); Cependant, ils ont observé une disparité en termes de communication avec l'espace sous-arachnoïdien. Acosta *et al.* [9] et Mummaneni *et al.* [20] ont observé que les patients qui présentent une douleur, exacerbées par les deux changements de posture et des manœuvres de Valsalva, et qui n'ont pas un dysfonctionnement urinaire, sont susceptibles de bénéficier le plus de la chirurgie.

**Tableau 2:** résultats des études dans la littérature dans lesquelles les patients ont bénéficié d’un traitement microchirurgical

Auteurs	Nombre des patients	Technique chirurgicale	Amélioration des symptômes	complications
Langdown *et al.* 2005 [14]	3	SL + CR + MP	100%	Fuite de LCS, délogement du patch musculaire
Giampaolo *et al.* 2013 [16]	19	SL+ CSFA +CWC	84,2%	non
Voyadzis *et al*. 2001 [17]	10	SL + CF +CI	87,5%	non
Mummaneni et al. 2000 [20]	8	SL + CF +CI	87,5%	non
Caspar *et al*. 2003 [21]	15	SL +PCR	86,7%	non
Neulen et al. 2011 [23]	13	SL + CF + CR	61,5%	Fuites de LCS
Guo *et al*. 2007 [26]	11	SL + PCR + CI	81,8%	Fuite de LCS, trouble urinaire
Park *et al*. 2008 [27]	2	SL + CR	100%	non
Sajko *et al*. 2009 [28]	3	SL + CF + CR	100%	non
Smith *et al.* 2011 [29]	18	SL$ + CF + FP	55,6%	Fuite du LCS, incontinence urinaire TVP
Tanaka *et al*. 2006 [30]	12	SRL + CR + CI	83,3%	Prostatite, hemorragie de la FCP
Notre étude	20	SL+PC+SD	80%	non

SL, sacral laminectomy; CF, cyst fenestration; CI, cyst imbrication; PCR, partial cyst resection; NL, neck ligation; CR, cyst resection; MP, muscle patch; CSFA, CSF aspiration; SRL, sacral recapping laminectomy; PF, posterior fossa; FP, fat packing; DVT, deep venous thrombosis; CWC, cyst wall clipping; PC, ponction cyst ; SD : sleeve of the dura ; $, Sacral laminotomy

## Conclusion

Notre technique chirurgicale (laminectomie, ponction du kyste à l'aide d'une aiguille fine et la mise en place d'un manchon dure-mèrien) semble efficace dans les 20 cas rapportés dans notre série. Cependant, la prise en charge des kystes de Tarlov nécessite une expérience clinique et chirurgicale bien mise en route. Au moment d'écrire ces lignes, une comparaison entre les différentes techniques chirurgicales est encore difficile, en raison du petit nombre de cas rapportés dans la littérature. La validation de nos résultats devrait être obtenue, par une étude multicentrique prospective.

### État des connaissances actuelles sur le sujet

- La prise en charge des kystes de Tarlov sacrés dépend du caractère communicant ou non dans la radiculosaccographie et l'érosion osseuse qui apparaît au scanner;
- Selon ces 2 caractéristiques radiologiques on choisit soit la technique mini-invasive qui est la ponction-aspiration du kyste et injection de la colle biologique à l'aide de deux aiguilles fines, soit la chirurgie à ciel ouvert dont les techniques sont différente et dépendent de chaque équipe et d'ailleurs il y a très peu d'équipe qui prennent en charge ce type de lésions

### Contribution de notre étude à la connaissance

- Notre technique chirurgicale n'est jamais décrite dans la littérature;
- Notre série semble la plus grande dans la littérature;
- Notre travail permet de faire une étude comparative avec les autres techniques chirurgicales, et permet aussi aux chirurgiens qui veulent prendre ou qui prennent en charge cette pathologie d'enrichir leurs savoir faire et pourquoi pas adopter notre technique dans l'avenir pour leurs patients surtout que nos résultats sont encourageantes.

## Conflits des intérêts

Les auteurs ne déclarent aucun conflit d'intérêts.
